# The expression of phospholipase A2 group X is inversely associated with metastasis in colorectal cancer

**DOI:** 10.3892/ol.2012.1067

**Published:** 2012-12-10

**Authors:** MASAYA HIYOSHI, JOJI KITAYAMA, SHINSUKE KAZAMA, YOSHITAKA TAKETOMI, MAKOTO MURAKAMI, NELSON H. TSUNO, KUMIKO HONGO, MANABU KANEKO, EIJI SUNAMI, TOSHIAKI WATANABE

**Affiliations:** 1 Division of Surgical Oncology, Department of Surgery, Faculty of Medicine, The University of Tokyo, Tokyo 113-8655;; 2 Lipid Metabolism Project, The Tokyo Metropolitan Institute of Medical Science, Tokyo 156-8506;; 3 Department of Transfusion Medicine, Faculty of Medicine, The University of Tokyo, Tokyo 113-8655, Japan

**Keywords:** phospholipase A2, colorectal cancer, immunohistochemistry, metastasis

## Abstract

Among the secretory phospholipase A2s (sPLA2), sPLA2 group X (PLA2GX) has the most potent hydrolyzing activity toward phosphatidylcholine, and has recently been shown to be implicated in chronic inflammatory diseases. The aim of the present study was to investigate PLA2GX expression in colorectal cancer (CRC) and its correlation with patient clinicopathological features. The present study comprises a series of 158 patients who underwent surgical resection for primary CRC. PLA2GX expression in CRC tissues was examined by immunohistochemistry and compared with patient clinicopathological findings and survival. A total of 64% of the tumors expressed PLA2GX at high levels. Statistical analysis revealed that PLA2GX expression was inversely correlated with hematogenous metastasis (P=0.005). In the subgroup analysis, left-sided tumors with high PLA2GX expression showed an inverse correlation with lymph node metastasis (P=0.018) and hematogenous metastasis (P=0.017). Patients with high PLA2GX expression tended to have a longer disease-specific survival compared with those with low PLA2GX expression in left-sided, but not right-sided, CRC (P=0.08). In light of the present results, we suggest that PLA2GX has an inhibitory effect on the progression of CRC.

## Introduction

Globally, colorectal cancer (CRC) is the third most commonly diagnosed cancer in males and the second in females (1). A number of studies have demonstrated the critical involvement of cyclooxygenase (COX) in the development and progression of CRC (2,3). COX is a rate-limiting enzyme in the synthesis of bioactive prostaglandins or thromboxanes from arachidonic acid (AA), which is mainly released from membrane-bound glycerophospholipids. Phospholipase A2 (PLA2) is a key esterase that cleaves the glycerophospholipids at the sn-2 ester bond to release a fatty acid and lysophospholipid (4). Therefore, the tissue expression of PLA2 is thought to have important roles in the development of CRC.

PLA2 proteins are broadly defined into three different classes: secretory PLA2 (sPLA2), cytosolic PLA2 (cPLA2) and Ca^2+^-independent PLA2 (iPLA2). Approximately one-third of the PLA2s belong to the sPLA2 family, which contains typically disulfide-rich, low molecular weight enzymes with strict Ca^2+^ dependence and a His-Asp catalytic dyad (5). To date, 11 sPLA2s (IB, IIA, IIC, IID, IIE, IIF, III, V, X, XIIA and XIIB) have been identified in mammals. After being secreted to the extracellular space, sPLA2s act on cellular membrane-bound phospholipids in an autocrine or paracrine manner, leading to the production of various inflammatory mediators, including prostaglandins, leukotrienes and thromboxane. The other cleavage products, namely lysophospholipids, such as lysophosphatidylcholine (LPC) and lysophosphatidic acid (LPA), also have various bioactivities. Moreover, sPLA2s also act on non-cellular phospholipids, including those in microvesicles, pulmonary surfactant, lipoproteins, microbial membranes and food substances (4).

The physiological functions of the different sPLA2s have been gradually elucidated. They have been implicated in lipid digestion and obesity, activation of immune cells, asthma, atherosclerosis, acute respiratory distress syndrome and host defense against bacteria, viruses and parasites (5–8). However, differences in pathophysiological roles as well as the expression profile of each enzyme remain largely unknown. Of the sPLA2 family, sPLA2 group X (PLA2GX) has the most powerful AA-releasing activity from cell membrane-bound phospholipids, leading to eicosanoid formation (9,10). Morioka *et al* have shown that PLA2GX also releases AA from cultured human colon carcinoma cell lines, leading to COX-2-dependent PGE2 formation (11). The authors also showed enhanced expression of PLA2GX in adenocarcinoma cells in comparison with the normal colonic epithelia, by immunohistochemistry. PLA2GX has also been shown to stimulate the proliferation of colon cancer cells (12). From these data, the positive role of PLA2GX on colorectal carcinogenesis is speculated. In fact, previous studies have also described the expression of PLA2GX in human colon cancer tissue at the mRNA (13) and protein (14) levels. However, the precise expression and distribution patterns of PLA2GX in colonic cancer tissues remain to be characterized. In the present study, we aimed to examine the expression of PLA2GX in human CRC tissue and its possible correlation with clinical and pathological variables as well as with patient outcome.

## Patients and methods

### Patients and samples

A total of 158 consecutive patients with colorectal adenocarcinoma who underwent curative resection with lymph node dissection at the University of Tokyo Hospital (Tokyo, Japan), in the period between January 1991 and March 1994, were enrolled. There were 96 males and 62 females (mean age, 62 years; range, 38–90 years). Cases of ulcerative colitis and familial adenomatous polyposis were excluded from this study. None of the patients had received preoperative chemotherapy or radiation therapy. All pertinent clinical and histopathological data of the patients and their tumors were collected from the patients’ case records. Clinicopathological features were analyzed based on the TNM classification of malignant tumors of the Union for International Cancer Control (UICC; 7th edition). All patients had been subsequently followed up at regular clinical visits until mortality or when last seen alive, for a mean observation period of 108 months. Informed consent was obtained from all patients and the study was approved by the Ethics Committee of the Hospital of the University of Tokyo, Tokyo, Japan.

The surgically resected specimens were immediately fixed in 10% buffered formalin and the cross-sections of the entire cancerous lesion were embedded in paraffin. Conventional pathological diagnosis of the primary lesion and the dissected lymph nodes was performed on hematoxylin and eosin (H&E)-stained sections. PLA2GX expression in the cancerous lesion was examined by immunohistochemical staining, as described below.

### Immunohistochemical study

Rabbit anti-sPLA2GX polyclonal antibody was generated by the immunization of rabbit with a polypeptide at The Tokyo Metropolitan Institute of Medical Science (Tokyo, Japan). The specificity and immunoreactivity of the antibody was verified by immunoblotting with sPLA2-transfected cells (15). Consecutive formalin-fixed paraffin-embedded sections (4 μm thick) were immunohistochemically stained by the streptavidin-biotin (SAB) immunoperoxidase method. For immunohistochemical staining, the sections were deparaffinized with xylene and dehydrated with 98% ethanol, placed in 0.01 M sodium citrate buffer (pH 6.0) and heated in an autoclave oven for 15 min. After washing twice in PBS, endogenous peroxidase activity was inhibited by incubation with 0.3% hydrogen peroxide in methanol for 20 min. After three washes in PBS, non-specific reactions were blocked by incubation with 10% goat serum for 30 min at room temperature. Biotinylated goat anti-rabbit immunoglobulin and SAB complex, supplied commercially [Histfine SAB-PO(R) kit, Nichirei, Tokyo, Japan] were used as the reagents in the subsequent steps. The sections were incubated with the anti-PLA2GX antibody overnight at 4°C. The color was then developed with diaminobenzidine solution. The sections were then lightly counterstained with a cocktail of Mayer’s/Lillie-Mayer’s hematoxylin and mounted. Spermatozoa were used as a positive control (16). For the negative control, the antibody was replaced with PBS.

### Evaluation of immunostaining

The expression of PLA2GX in the cancerous lesion and in the surrounding normal mucosa was assessed by two observers (S.K. and M.H.) without knowledge of the corresponding clinical data. All tissue samples were assessed in a consecutive analysis to ensure maximal internal consistency. For the objective assessment of the PLA2GX expression level, it was stratified into three groups, as follows: −, not detected; +, focally positive in cancer cells; ++, diffusely positive in carcinoma cells. The consistency between the observers was 80.1% (κ test), and in the discrepant cases, a consensus was reached after a joint review. In the statistical analysis, − and + were considered to be the low expression group, and ++ was considered to be the high expression group.

### Statistical analysis

The statistical significance of the differences was evaluated by χ^2^ test, Fisher’s exact test or a non-paired Student’s t-test, as appropriate. The disease-specific survival (DSS) rate was analyzed by the Kaplan-Meier method and the log-rank comparison test. To assess the value of PLA2GX as an independent predictor, a multivariate survival analysis was performed, using the Cox proportional hazards regression model. All statistical analyses were performed with JMP 9.0 (SAS Institute, Cary, NJ, USA). P<0.05 was considered to indicate a statistically significant result.

## Results

### PLA2GX expression in human CRC

**Figure 1.**
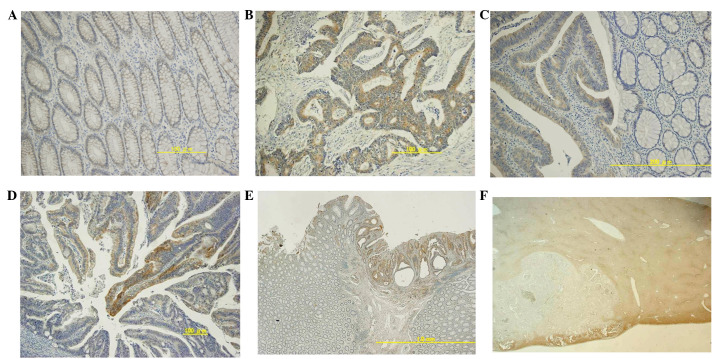
Expression pattern of PLA2GX in CRC specimens and normal counterparts. Staining in (A) the normal colonic mucosa (weak staining) ; (B) the cancer tissue (diffuse staining); (C) the boundary area between normal (right) and cancer (left); and (D) the cancer tissue (focal immunoreactivity). Representative cases of (E) primary CRC and (F) hepatic metastasis. PLA2GX, secretory phospholipase A2 group X; CRC, colorectal cancer. (A and B) Magnification, ×200. (C–E) Magnification, ×100. (F) Magnification, ×20.

The staining patterns of PLA2GX in CRC specimens are shown in Fig. 1. In the normal colonic mucosa adjacent to the CRC, most of the colonic epithelial cells showed a weak expression of PLA2GX (Fig. 1A), although there was a variation of immunoreactivity among the cases. In the majority of the 158 tumors, PLA2GX expression was found predominantly in the cytoplasm of carcinoma cells and, compared with the normal epithelium, the staining signal was generally enhanced (Fig. 1B). The stromal tissue was not stained in any analyzed specimen. In 101 cases, PLA2GX expression was diffusely and almost equally detected in most of the cancer cells, as shown in Fig. 1B, whereas in 54 cases, the expression was observed only in focal cancer cells (Fig. 1D). In 3 cases, however, negligible staining of cancer cells was found. In addition, PLA2GX was hardly detected in the hepatic metastatic lesions, although the expression was diffuse in the primary lesion. (Fig. 1E and F). For further analyses, the tumors were divided into high expression (101 cases) and low expression (57 cases) groups.

### Correlation between PLA2GX expression and clinicopathological features

Descriptive characteristics of the study subjects are presented in Table I. The expression of PLA2GX showed no correlation with age, gender, tumor size, tumor location, histological appearance, lymphatic invasion or venous involvement. However, the rate of hematogenous metastasis was significantly higher in the low PLA2GX expression group (10.5%) than in the high PLA2GX expression group (1.0%; P= 0.005). Similarly, there was a tendency for higher incidence of lymph node metastasis in the low PLA2GX expression group (50.9%) than in the high expression group (36.6%; P=0.081) (Table II). When the study sample was restricted to left-sided tumors (112 cases), nodal metastasis was observed in 52.4% (22/42) of the low PLA2GX expression group, which was significantly higher than that in the high expression group (30.0%; 21/70; P= 0.018). By contrast, when samples were restricted to right-sided tumors, no difference in the incidence of nodal metastasis was observed. Additionally, only border-line significance (P=0.051) was observed in the association between the PLA2GX expression and the UICC stage.

### Overall survival and DSS analysis of CRC with regard to PLA2GX expression

**Figure 2.**
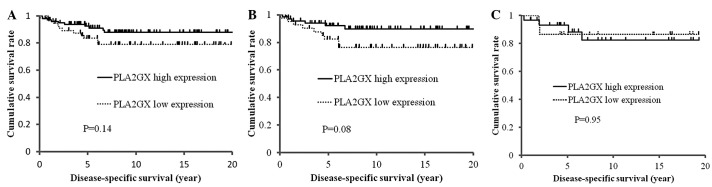
Survival outcomes of the patients grouped according to the expression of PLA2GX. Kaplan-Meier estimates of disease-specific survival of (A) all CRC patients, and those of (B) left- and (C) right-sided colon cancer. PLA2GX, phospholipase A2 group X.

Next, we examined the correlation between PLA2GX expression and the outcome of patients, by the Kaplan-Meier analysis and the log-rank test. As shown in Fig. 2A, the high PLA2GX expression group tended to have a longer DSS, although the difference did not reach statistical significance (P=0.14). This trend was pronounced in left-sided CRC (P=0.08) and was observed in right-sided CRC (Fig. 2B).

## Discussion

Due to their functional diversity, PLA2 enzymes have been implicated in various biological processes, including arthritis, asthma, defense against microbes, digestion, atherosclerosis and cancer (17). PLA2GX is known as the most potent sPLA2 capable of hydrolyzing phosphatidylcholine and acting extra-cellularly on cellular membranes and noncellular phospholipid substrates (5,9–11). It has been confirmed that PLA2GX, as well as other sPLA2s, including GIIA, GIII and GXIIA, are highly expressed in CRC tissue (13,14,18). However, no information is available concerning the correlation between PLA2GX expression in cancer and clinicopathological features. Thus, in the present study, we aimed to investigate the expression of PLA2GX in patients with CRC at various stages.

In our series, the majority of the carcinoma cells in primary CRC showed enhanced cytoplasmic expression of PLA2GX as compared with normal colonic epithelia, which is consistent with previous results (11,13,14). However, in 64% of the 158 cases, PLA2GX expression was diffusely detected, while in certain cases PLA2GX was only partially expressed and in a few cases, its expression was negative. In these cases, a significant inverse correlation was found between PLA2GX expression and hematogenous metastasis and, although without significance, also with nodal metastasis. As a consequence, patients with tumors with high PLA2GX expression had a better outcome than those with low expression. To the best of our knowledge, this is the first study to show the inverse correlation of the expression of PLA2GX with the outcome of CRC patients, and the possibility that this enzyme has suppressive effects on tumor metastasis in CRC is suggested.

Increased expression of sPLA2 has been demonstrated in numerous types of cancer, including breast (19,20), pancreatic (21), prostate (22,23), liver (24), gastric (25,26) and colorectal (14,27) cancer. Of these, group IIA PLA2 (PLA2GIIA) is one of the isoforms most commonly investigated in cancer tissue. Kashiwagi *et al* reported that the high expression of PLA2GIIA was correlated with longer survival in pancreatic cancer (28). More recently, Xing *et al* have shown the same tendency in gastric cancer, and suggested that PLA2GIIA may negatively affect the meta-static potential of gastric cancer cells (26). In fact, Ganesan *et al* have demonstrated that the silencing of the PLA2GIIA gene enhances the invasive activity of tumor cells, whereas enforced expression inhibited invasion (29). These results are consistent with our finding on PLA2GX, suggesting a possibility that PLA2GX, as well as PLA2GIIA, may play negative roles in the metastatic potential of gastrointestinal cancer. However, Buhmeida *et al* have shown a negative association of the expression of PLA2GIIA with the prognosis of stage II CRC (27). Also, Graff *et al* have shown that PLA2GIIA expression increases with the progression of prostate cancer in an androgen-independent manner (30). These controversial results suggest that the role of PLA2GIIA is dependent on the interaction of cancer cells with the microenvironment in different tissues.

Surrel *et al* have reported that, *in vitro*, the addition of PLA2GX stimulates the proliferative ability of colon cancer cells by producing various lipid mediators (12). However, in their study, the proliferation of the cells was examined in serum-free media, and the effect was not completely abrogated by COX and lipooxygenase inhibitors, suggesting the involvement of other lipid mediators. The released AA itself induces the apoptosis of diverse cells, including human colon cancer cells, via an intracellular ceramide-mediated pathway (31). Ceramide acts as a second messenger in the activation process of the cellular apoptotic machinery (31). Diets rich in unsatu-rated fatty acids such as AA are associated with a decreased incidence of colon cancer (32). On the bases of these facts, the negative effect of PLA2GX may be attributed to the increased levels of AA produced and the subsequent increased susceptibility to apoptosis.

Although there is no significant difference between PLA2GX expression and the tumor location, the rate of lymph node metastasis was found to be significantly lower and the DSS better in left-sided CRC than in right-sided. This finding suggests the possibility that CRCs developing in the two different colonic sites may have different biological behaviors. It is known that right- and left-sided colonic tumors have different molecular profiles, with microsatellite instability and methylator phenotypes being prevalent in right-sided tumors and chromosomal instability being predominant in left-sided tumors, resulting in different biological features (33,34). It is possible that these molecular profiles are intimately associated with the different lymphatic metastatic potentials. Indeed, the PLA2GX gene expression level was reported to be significantly higher in the normal mucosa of the left side of the colon than in that of the right side (13).

In conclusion, our study showed an inverse association between the reduced expression of PLA2GX and the increased metastatic potential of human CRC, especially in the left-sided tumors. Our data suggest that PLA2GX may have a protective role against the invasive ability of CRC, and the reduced ability to produce PLA2GX may result in the acquisition of a clinically more malignant phenotype. Thus, PLA2GX expression may be a potential clinical biomarker for the prediction of the invasive ability of human CRC. To date, several inhibitors of PLA2s have been developed as anticancer drugs (35). However, since PLA2s, in certain situations, may also have a suppressive effect on tumor progression, they may be two-edged swords, and thus their indication should be carefully considered.

## Figures and Tables

**Table I.** Association of PLA2GX expression with clinical variables.

		PLA2GX		
Factor	n	High expression (n=101)	Low expression (n=57)	P-value
Age (years), mean ± SD	158	62.4±11.1	63.6±10.3	0.481
Gender, n (%)				
Male	96	41 (40.6)	21 (36.8)	0.642
Female	62	60 (59.4)	36 (63.2)	
Size of tumor (mm), mean ± SD	158	46.5±24.1	47.3±17.7	0.824
T stage^a^, n (%)				
T1/T2	36	27 (26.7)	9 (15.8)	0.108
T3/T4	122	74 (73.3)	48 (84.2)	
Histological type, n (%)				
Well, mod differentiated	152	96 (95.0)	56 (98.2)	0.285
Muc, por differentiated	6	5 (5.0)	1 (1.8)	
Lymphatic invasion, n (%)				
Absent	118	76 (75.2)	42 (73.7)	0.829
Present	40	25 (24.8)	15 (26.3)	
Lymph node metastasis, n (%)				
Absent	92	64 (63.4)	28 (49.1)	0.081
Present	66	37 (36.6)	29 (50.9)	
Venous involvement, n (%)				
Absent	75	50 (49.5)	25 (43.9)	0.495
Present	83	51 (50.5)	32 (56.1)	
Location of the tumor, n (%)				
Colon	117	78 (77.2)	39 (68.4)	0.229
Rectum	41	23 (22.8)	18 (31.6)	
Right side	46	31 (30.7)	15 (26.3)	0.559
Left side	112	70 (69.3)	42 (73.7)	
UICC stage, n (%)				
I/II	91	64 (63.4)	27 (47.4)	0.051
III/IV	67	37 (36.6)	30 (52.6)	
Hematogenous metastasis, n (%)				
Absent	151	100 (99.0)	51 (89.5)	0.005
Present	7	1 (1.0)	6 (10.5)	

UICC, Union for International Cancer Control;

a TMN classification of malignant tumors, 7th edition according to UICC. PLA2GX, secretory phospholipase A2 group X; muc, mod, mucinous; moderately; por, poorly.

**Table II.** Association of PLA2GX expression in the left side of the colon with clinical variables.

		PLA2GX, n (%)		
Factor	n	High expression (n=70)	Low expression (n=42)	P-value
Lymphatic invasion				
Absent	85	54 (77.1)	31 (73.8)	0.691
Present	27	16 (22.9)	11 (26.2)	
Lymph node metastasis				
Absent	69	49 (70.0)	20 (47.6)	0.018
Present	43	21 (30.0)	22 (52.4)	
Venous involvement				
Absent	54	35 (50.0)	19 (45.2)	0.625
Present	58	35 (50.0)	23 (54.8)	
Hematogenous metastasis				
Absent	105	69 (98.6)	36 (85.7)	0.017
Present	7	1 (1.4)	6 (14.3)	

PLA2GX, secretory phospholipase A2 group X.
